# Development of a coordinated interhospital transfer program for cardiac surgery patients

**DOI:** 10.1186/s13019-025-03641-1

**Published:** 2025-10-27

**Authors:** Jenkins FS, Yilmaz E., Vallejo Castano LJ, Bektas B., Najdawi A., Dalyanoglu I., Bayer N., Lichtenberg A., Dalyanoglu H.

**Affiliations:** 1 Department of Cardiac Surgery, Medical Faculty, Heinrich Heine University, Dusseldorf, Germany; 2 Department of Cardiology, St.-Antonius-Hospital Kleve, Kleve, Germany; 3 Medical Faculty, Semmelweis University, Budapest, Hungary

**Keywords:** Cardiac surgery, Interhospital transfer, Emergent surgery, Elective surgery, Urgent surgery, Patient outcomes

## Abstract

**Background:**

Timely access to specialized cardiac surgical care is essential for optimal outcomes in patients with complex cardiovascular conditions. Interhospital transfer (IHT) programs have the potential to bridge the gap between regional hospitals and tertiary centers. This study evaluates the establishment of a structured collaboration between a district hospital and a university medical center, with a coordinated interhospital transfer (IHT) program as a key component. Patient characteristics, transfer logistics, and clinical outcomes across elective, urgent, and emergent admissions were analyzed. Despite the presence of other tertiary centers in the region, the referring hospital consistently transferred patients to our center, which has become its sole cardiac surgical provider within this cooperation.

**Methods:**

A retrospective cohort study was conducted including 793 patients transferred between January 2018 and March 2023. Patients were classified based on clinical urgency as elective (*n* = 240), urgent (*n* = 379), or emergent (*n* = 174). Data collected included demographics, comorbidities, ASA classification, surgical type, preoperative risk factors, transfer times, time from admission to surgery, and in-hospital mortality. Comparative analyses used Chi-squared, Kruskal-Wallis, and Mann-Whitney U tests. Kaplan-Meier curves and ROC analysis were performed for survival and time-to-surgery impact.

**Results:**

Emergent patients were significantly more often classified as ASA class 4 (74%) and had higher rates of preoperative myocardial infarction (55%), shock (16%), and CPR (5.2%) compared to urgent and elective patients. CABG was the predominant procedure (69%), especially among emergent cases (80%). Time from admission to surgery was significantly shorter for emergent patients (median 4 h) compared to urgent (25 h) and elective (75 h). In-hospital mortality was highest in emergent patients (6.9%, *p* = 0.002). ROC analysis did not reveal a predictive threshold for time-to-surgery.

**Conclusion:**

A coordinated IHT program facilitates timely cardiac surgical care, particularly for high-risk emergent cases. Further refinement of triage criteria and integration of telemedicine may enhance program efficacy.

## Introduction

 Interhospital transfer (IHT) programs are essential for ensuring that patients with complex cardiac conditions receive timely access to specialized surgical care [1, 2]. Acute coronary syndromes, aortic dissections, severe valvular disease, and heart failure syndromes often present at community or district hospitals that lack the infrastructure for advanced cardiac surgical interventions [3, 4]. Consequently, rapid and organized transfer to tertiary centers is critical for optimizing outcomes [5, 6]. This study describes the establishment of a general cooperation as well as the complementary implementation and evaluation of a structured interhospital transfer (IHT) program developed between the University Hospital Düsseldorf and the Karl Leissner Clinic in Kleve, located approximately 100 km apart. Initiated through collaborative agreements between institutional leadership, the program aimed to standardize triage, transport, and perioperative care for patients requiring elective, urgent, or emergent cardiac surgery. Over a five-year period, the program facilitated the transfer of nearly 800 patients, providing a comprehensive dataset to analyze surgical urgency, preoperative risk, and outcomes. The types of cardiac surgeries performed as part of this program included coronary artery bypass grafting (CABG), aortic and mitral valve replacements, and less frequently, complex aortic and mixed procedures. Emergent transfers primarily involved patients with acute myocardial infarction, cardiogenic shock, or hemodynamic instability, necessitating CABG or urgent valve surgery. Urgent patients often required expedited care within 24–48 h due to symptomatic but stable conditions, whereas elective patients underwent planned procedures We hypothesize that a coordinated IHT program improves timely access to cardiac surgery, especially in high-risk emergent cases. Assessing these categories provides insight into the program’s effectiveness in handling different clinical scenarios.

## Methods

### Study design and population

This study employed a retrospective cohort design to evaluate perioperative outcomes among patients transferred for cardiac surgery under a coordinated interhospital transfer program between a regional district hospital and a tertiary care center. Patients were identified by cross-referencing institutional databases from the participating hospitals, specifically the Department of Cardiac Surgery at the University Hospital Düsseldorf and the Karl-Leisner-Klinikum Kleve. All patients transferred between January 2018 and March 2023 were included. A total of 793 patients were categorized based on urgency: elective (*n* = 240), urgent (*n* = 379), and emergent (*n* = 174). Elective cases involved planned admissions for non-urgent procedures, urgent cases required intervention within 24 to 72 h, and emergent cases required immediate intervention due to life-threatening conditions.

### Data collection

Patient data were extracted from institutional electronic health records. Demographics (age, sex, BMI), comorbidities (history of myocardial infarction [MI], CPR, or shock), and preoperative markers (ventilation, positive troponin), nicotine, arterial hypertension, insulin-dependent and non-insulin-dependent diabetes mellitus, cerebral arterial occlusive disease, peripheral arterial occlusive disease, previous cardiac surgery, preoperative renal replacement therapy, and previously known COPD were recorded. ASA (American Society of Anesthesiologists) classification was used to assess surgical risk. Surgical variables included type of procedure (CABG, aortic valve replacement, mitral valve surgery, or mixed procedures), surgery duration, and the time from hospital admission to surgical intervention. Transport logistics were documented, including time from transfer request to arrival.

### Statistical analysis

Categorical variables were compared using Pearson’s Chi-squared or Fisher’s exact tests, as appropriate. Continuous variables were evaluated using Kruskal-Wallis and Mann-Whitney U tests due to non-normal distributions. Kaplan-Meier survival analysis was used to evaluate 90-day survival differences by admission category, with the log-rank test for significance. ROC curve analysis was used to assess whether time between hospital admission and surgery predicted mortality in emergent patients. All analyses were performed using R (version 4.2.1), with significance set at *p* < 0.05.

## Results

### Baseline characteristics

Among 793 patients, 75% were male, with a median age of 68 years (IQR 61–75). (See Table 1). Age distribution was comparable across admission types. Emergent patients exhibited significantly higher clinical acuity. CPR within 48 h was reported in 5.2% of emergent patients, compared to 0.3% of urgent and 0% of elective cases (*p* < 0.001). Similarly, recent MI (within 48 h) was more frequent in emergent patients (55%) than in urgent (6.1%) or elective (0.8%) groups (*p* < 0.001). ASA classification reflected increasing acuity, with 74% of emergent patients categorized as ASA class 4 or higher. Troponin positivity was highest among emergent cases (83%).

**Table 1:** Characteristics by admission type (*n* = 793)

Variable^1^	All patients *N* = 793	Elective *N* = 240	Urgent *N* = 379	Emergent *N* = 174	*p*-value^2^
**Sex**					0.4
Male	597 (75%)	177 (74%)	282 (74%)	138 (79%)	
Female	196 (25%)	63 (26%)	97 (26%)	36 (21%)	
**BMI**	28 (25, 31)	28 (25, 31)	27 (24, 31)	28.0 (25, 31)	0.3
**Age in years**	68 (61, 75)	69 (62, 76)	68 (61, 75)	65 (59, 74)	0.11
**CPR last 48 h**	10 (1.3%)	0 (0%)	1 (0.3%)	9 (5.2%)	**< 0.001**
**CPR ever**	18 (2.3%)	2 (0.8%)	5 (1.3%)	11 (6.3%)	**< 0.001**
**MI last 48 h**	121 (15%)	2 (0.8%)	23 (6.1%)	96 (55%)	**< 0.001**
**MI ever**	230 (29%)	44 (18%)	79 (21%)	107 (61%)	**< 0.001**
**ASA class**					**< 0.001**
2	6 (0.8%)	3 (1.3%)	2 (0.5%)	1 (0.6%)	
3	578 (73%)	210 (88%)	325 (86%)	43 (25%)	
4	208 (26%)	27 (11%)	52 (14%)	129 (74%)	
5	1 (0.1%)	0 (0%)	0 (0%)	1 (0.6%)	
**Ventilated**	41 (5.2%)	10 (4.2%)	13 (3.4%)	18 (10%)	**0.002**
**Shock last 48 h**	35 (4.4%)	3 (1.3%)	5 (1.3%)	27 (16%)	**< 0.001**
**Shock ever**	109 (14%)	29 (12%)	45 (12%)	35 (20%)	**0.022**
**High troponin**	485 (61%)	123 (51%)	217 (57%)	145 (83%)	**< 0.001**
**Surgery type**					n/a
Aorta	13 (1.6%)	2 (0.8%)	4 (1.1%)	7 (4.0%)	
Aortic valve	116 (15%)	42 (18%)	63 (17%)	11 (6.3%)	
CABG	549 (69%)	158 (66%)	251 (66%)	140 (80%)	
Mitral valve	83 (10%)	29 (12%)	49 (13%)	5 (2.9%)	
Other	32 (4.0%)	9 (3.8%)	12 (3.2%)	11 (6.3%)	
**Surgery duration**	220 (171, 269)	224 (172, 275)	222 (176, 269)	208 (164, 260)	0.2
**Adm. to surgery (h)**	27 (20, 71)	75 (53, 113)	25 (23, 31)	4 (2, 9)	**< 0.001**
**In-hospital mortality**	22 (2.8%)	3 (1.3%)	7 (1.8%)	12 (6.9%)	**0.002**

^1^Median (Q1, Q3)

^2^Pearson’s Chi-squared test; Kruskal-Wallis rank sum test; Fisher’s exact test

Legend: BMI = Body mass index; CPR = Cardiopulmonary resuscitation; MI = Myocardial infarction; ASA = American Society of Anesthesiologists; CABG = Coronary artery bypass grafting; Adm = Admission at recipient hospital. Significant p-values are highlighted in bold

### Surgical procedures and timing

**Fig. 1:**
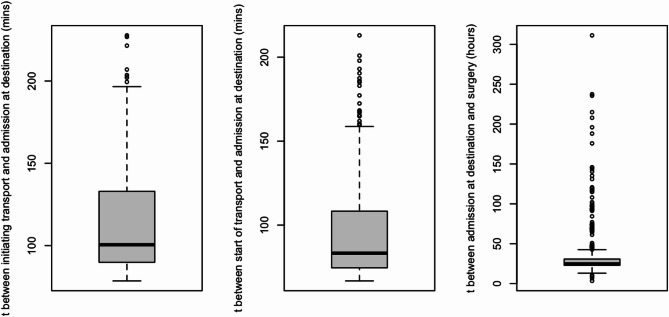
Time durations for urgent patient transfers (n=379)

**Fig. 2:**
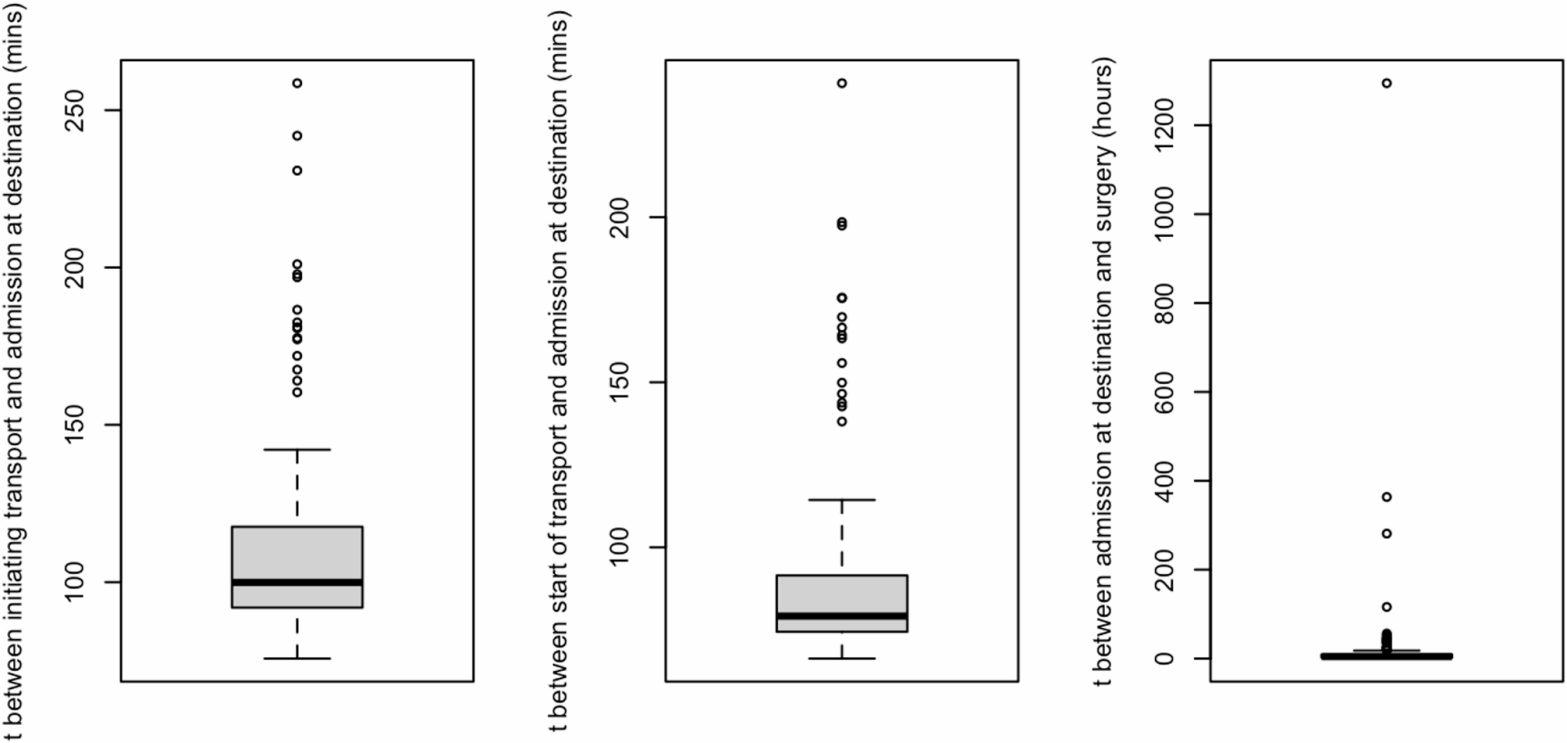
Time durations for emergent patient transfers (n=174)

CABG was the most common procedure (69% overall), particularly prevalent in emergent patients (80%). Aortic valve surgeries (15%) and mitral valve interventions (10%) were more frequent among elective and urgent patients. An additional 4.0% of procedures were categorized as “other”, including tricuspid valve surgeries, left atrial tumor resections, pericardiectomies, atrial septal defect (ASD) closures, and heart transplantations. Median surgery duration did not differ significantly between groups (*p* = 0.2). Time from admission to surgery was shortest in emergent cases (median 4 h, IQR 2–9), significantly shorter than urgent (25 h, IQR 23–31) and elective (75 h, IQR 53–113) patients (*p* < 0.001) (Figs. 1 and 2). Median time from transfer call to arrival was 90 min (IQR 70–130) across urgent and emergent patients. Actual transport duration (from departure to arrival) had a median of 70 min (IQR 50–110). Timing metrics were consistent over the study period.

### Temporal trends

Urgent admissions increased over time, peaking at 53% in 2022. Emergent cases remained relatively stable across years (range 19–28%) with a trend towards more emergent cases over time. (See Table 2). The proportion of ventilated patients increased over time, notably in 2022 (13%), suggesting rising acuity. CABG consistently remained the predominant surgery annually.

**Table 2:** Patient characteristics by year of interhospital transfer program

Variable^1^	2018 *N* = 70	2019 *N* = 202	2020 *N* = 197	2021 *N* = 165	2022 *N* = 127	2023 *N* = 32
**Admission type**						
Elective	24 (34%)	72 (36%)	47 (24%)	52 (32%)	35 (28%)	10 (31%)
Urgent	31 (44%)	92 (46%)	101 (51%)	75 (45%)	67 (53%)	13 (41%)
Emergent	15 (21%)	38 (19%)	49 (25%)	38 (23%)	25 (20%)	9 (28%)
**ASA class**						
2	1 (1.4%)	3 (1.5%)	1 (0.5%)	0 (0%)	1 (0.8%)	0 (0%)
3	50 (71%)	156 (77%)	129 (65%)	119 (72%)	102 (80%)	22 (69%)
4	19 (27%)	42 (21%)	67 (34%)	46 (28%)	24 (19%)	10 (31%)
5	0 (0%)	1 (0.5%)	0 (0%)	0 (0%)	0 (0%)	0 (0%)
**Ventilated**	4 (5.7%)	7 (3.5%)	1 (0.5%)	10 (6.1%)	17 (13%)	2 (6.3%)
**Shock last 48 h**	5 (7.1%)	6 (3.0%)	9 (4.6%)	9 (5.5%)	4 (3.1%)	2 (6.3%)
**Positive troponin**	46 (66%)	135 (67%)	119 (60%)	97 (59%)	68 (54%)	20 (63%)
**Surgery type**						
Aorta	1 (1.4%)	3 (1.5%)	5 (2.5%)	2 (1.2%)	1 (0.8%)	1 (3.1%)
Aortic valve	12 (17%)	31 (15%)	23 (12%)	24 (15%)	22 (17%)	4 (13%)
CABG	42 (60%)	145 (72%)	138 (70%)	116 (70%)	85 (67%)	23 (72%)
Mitral valve	10 (14%)	18 (8.9%)	23 (12%)	18 (11%)	12 (9.4%)	2 (6.3%)
Other	5 (7.1%)	5 (2.5%)	8 (4.1%)	5 (3.0%)	7 (5.5%)	2 (6.3%)

^1^Median (Q1, Q3)Legend: ASA=American Society of Anesthesiologists; CABG=Coronary artery bypass grafting

### Mortality rates

The in-hospital mortality rate was significantly higher in emergent patients (6.9%) compared to elective (1.3%) and urgent (1.8%) patients (*p* = 0.002). Longitudinal survival for the first ninety days after surgery was also significantly higher in emergent cases (See Table 1).  The 12 patients admitted as emergencies who died in the hospital had a significantly higher prevalence of CPR in the last 48 h (25%) compared to those who survived to discharge (3.7%, *p* = 0.017). (See Table 3). Additionally, more emergent patients who died had a history of shock (58%) compared to those who survived (17%, *p* = 0.003). Ventilation at admission was significantly more common patients who died (42% vs. 8%, *p* = 0.003). The surgical characteristics of emergent patients who survived and died were similar. Surgery duration and the time from admission to surgery were not significantly different between the groups. The most common procedures in both groups were CABG (81% of survivors and 75% of non-survivors), followed by aortic valve surgeries (6.8% of survivors and 0% of non-survivors). There were no significant differences in surgery duration or the time to surgery (all *p* > 0.05).

**Table 3:** In-hospital mortality in patients transferred as emergent (*n* = 174)

Variable^1^	All emergencies *N* = 174	Survived discharge *N* = 162	Died in hospital *N* = 12	*p*-value^2^	
**Sex**				0.7	
Male	138 (79%)	129 (80%)	9 (75%)		
Female	36 (21%)	33 (20%)	3 (25%)		
**BMI**	28.0 (25.0, 31.0)	28.0 (25.0, 31.0)	26.5 (24.5, 32.5)	0.9	
**Age in years**	65 (59, 74)	65 (59, 74)	66 (57, 78)	0.9	
**CPR last 48 h**	9 (5.2%)	6 (3.7%)	3 (25%)	**0.017**	
**CPR ever**	11 (6.3%)	7 (4.3%)	4 (33%)	**0.003**	
**MI last 48 h**	96 (55%)	87 (54%)	9 (75%)	0.2	
**MI ever**	107 (61%)	97 (60%)	10 (83%)	0.13	
**ASA class**				**0.015**	
2	1 (0.6%)	1 (0.6%)	0 (0%)		
3	43 (25%)	43 (27%)	0 (0%)		
4	129 (74%)	118 (73%)	11 (92%)		
5	1 (0.6%)	0 (0%)	1 (8.3%)		
**Ventilated**	18 (10%)	13 (8.0%)	5 (42%)	**0.003**	
**Shock last 48 h**	27 (16%)	22 (14%)	5 (42%)	**0.023**	
**Shock ever**	35 (20%)	28 (17%)	7 (58%)	**0.003**	
**High troponin**	145 (83%)	133 (82%)	12 (100%)	0.2	
**Surgery type**				0.2	
Aorta	7 (4.0%)	7 (4.3%)	0 (0%)		
Aortic valve	11 (6.3%)	11 (6.8%)	0 (0%)		
CABG	140 (80%)	131 (81%)	9 (75%)		
Mitral valve	5 (2.9%)	4 (2.5%)	1 (8.3%)		
Other	11 (6.3%)	9 (5.6%)	2 (17%)		
**Surgery duration**	208 (164, 260)	204 (162, 255)	261 (196, 295)	0.15	
**Adm. to surgery (h)**	4 (2, 9)	4 (2, 9)	4 (1, 9)	0.6	

^1^Median (Q1, Q3)

^2^Pearson’s Chi-squared test; Kruskal-Wallis rank sum test; Fisher’s exact test

Legend: BMI = Body mass index; CPR = Cardiopulmonary resuscitation; MI = Myocardial infarction; ASA = American Society of Anesthesiologists; CABG = Coronary artery bypass grafting; Adm = Admission at recipient hospital. Significant p-values are highlighted in bold

**Fig. 3:**
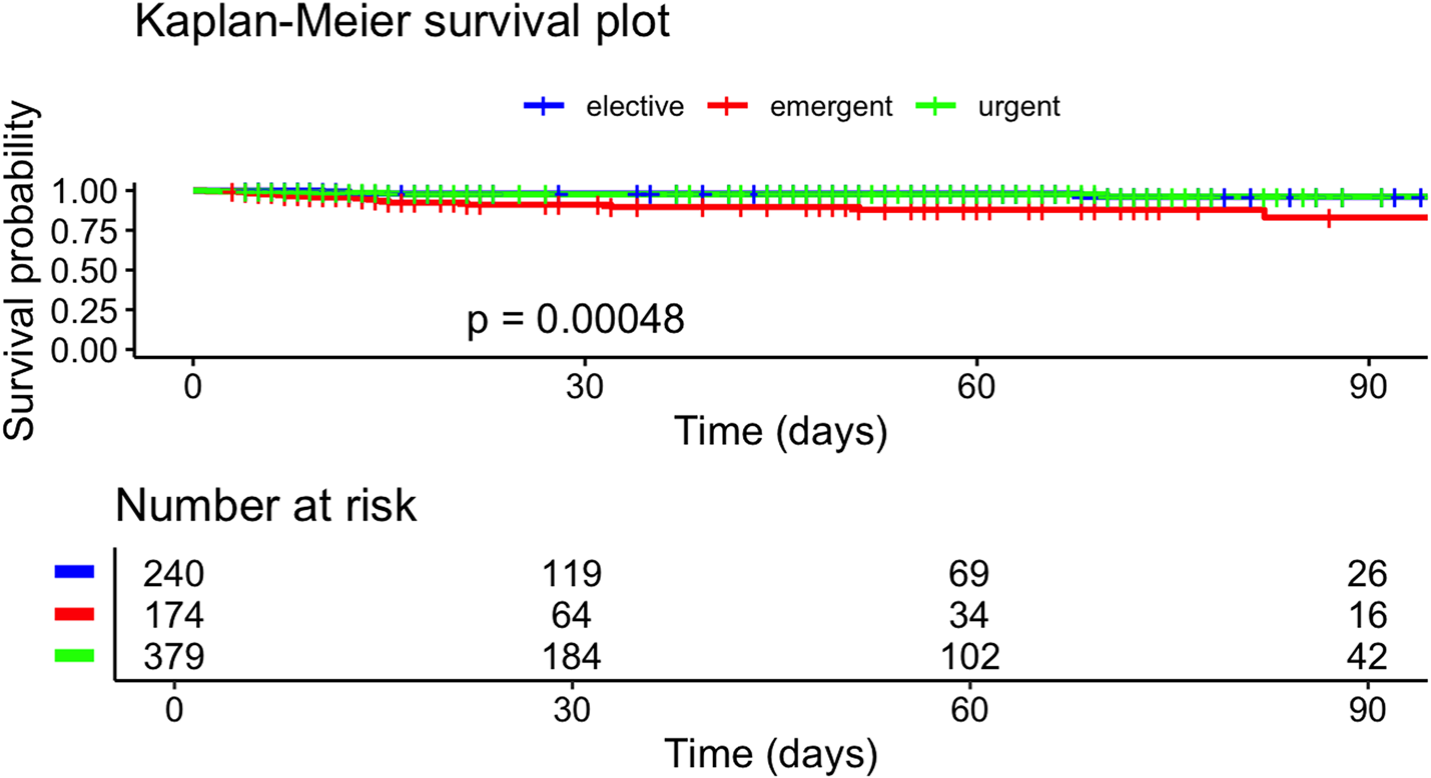
Kaplan-Meier survival plot by type of admission (ninety days after surgery)

The Kaplan-Meier survival analysis demonstrated a statistically significant difference in 90-day survival rates based on the type of hospital admission (elective, emergent, or urgent) (Fig. 3) (*p* = 0.00048). 

Elective admissions exhibited the highest survival probability, while emergent admissions showed the lowest. Urgent admissions had an intermediate survival rate (See Fig. 3).

**Fig. 4:**
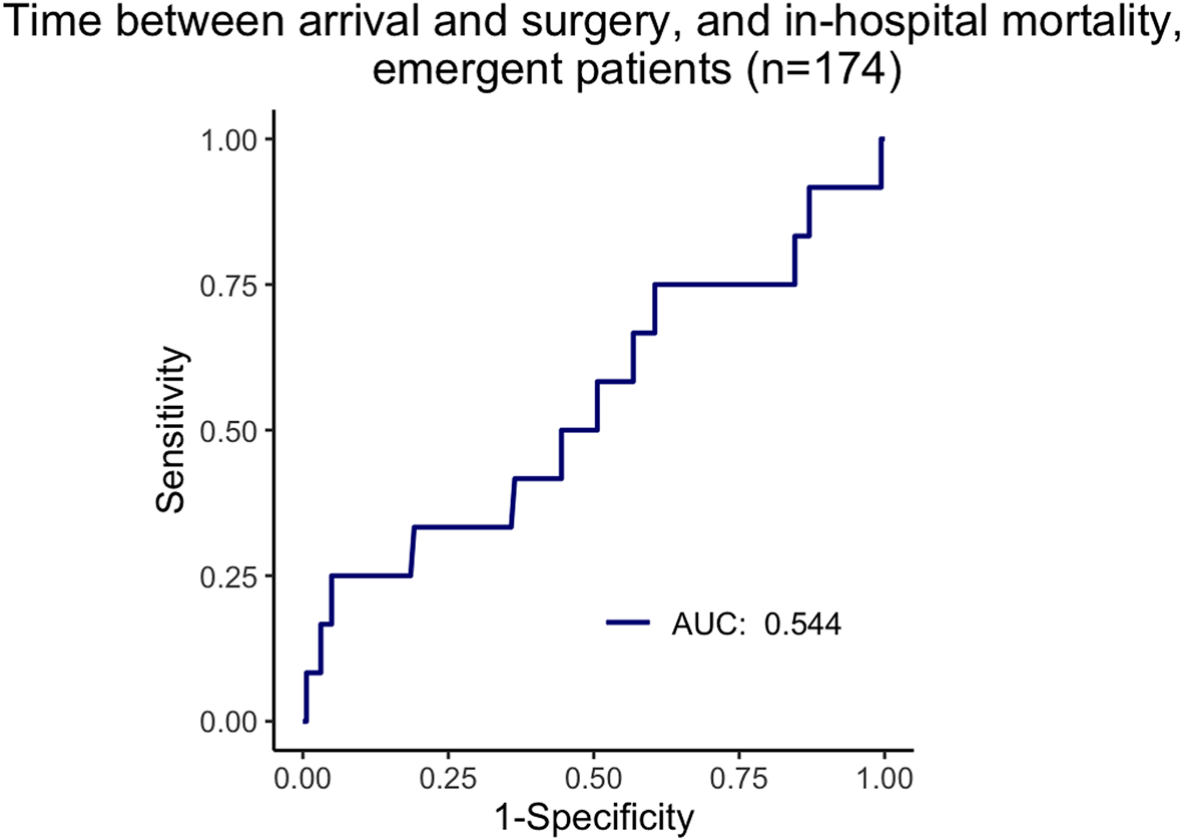
ROC curve for time between arrival and start of surgery, and in-hospital mortality for patients transferred as emergencies

Time to surgery was also not a significant discriminator of in-hospital in ROC curve analysis (See Fig. 4).

## Discussion

This study underscores the efficacy of a structured interhospital transfer program in managing patients requiring various levels of cardiac surgical intervention. Key findings reveal distinct patterns in patient characteristics, urgency classifications, and clinical outcomes, similar findings were found in a study from Sanaiha et al. [7]. Emergent patients displayed significantly higher rates of myocardial infarction, preoperative shock, and mechanical ventilation, which correlated with an elevated in-hospital mortality rate of 6.9%, findings consistent with these were reported by Metkus [8]. These results were not surprising, as those criteria are, per se, predictors of in-hospital mortality in the STS Score and the EuroSCORE II [9, 10]. In contrast, urgent and elective patients had lower mortality rates and reduced preoperative risk profiles. At Düsseldorf University Hospital, we operated on an average of 1,650 patients per year. The overall in-hospital mortality rate was 5.2%. Stratified by urgency, the mortality rate was 2.0% for elective cases, 5.2% for urgent cases, and 16% for emergency cases. The markedly high mortality rate among emergency patients can be attributed, among other factors, to the high proportion of in-house resuscitations from other departments within the university hospital, as well as to the frequent admissions following out-of-hospital cardiac arrests, given our role as a major cardiac arrest center. Interestingly, the emergency patient cohort transferred from the Karl-Leisner-Klinikum demonstrated a notably lower in-hospital mortality rate. This discrepancy may be explained by significant differences in patient selection and timing. In particular, the patients referred emergently from external hospitals were usually hemodynamically stabilized prior to transport, and resuscitated patients were typically not transferred unless a return of spontaneous circulation (ROSC) had been achieved and a certain degree of neurological prognosis could be anticipated. Additionally, the Karl-Leisner-Klinikum lacks a dedicated cardiac arrest center, which may have led to a different triage and referral strategy, focusing more on potentially salvageable patients with a better short-term prognosis. Consequently, while the clinical severity of transferred patients was still high, the proportion of high-risk, non-survivable emergencies may have been lower compared to the in-house emergency population at the tertiary center.

The stepwise development of the program allowed for iterative improvements in communication, triage, and transport logistics [11]. Trends across the five-year period demonstrated a gradual shift toward a higher proportion of urgent admissions, reflecting growing confidence and reliance on the transfer protocol by referring clinicians. The stability of emergent case proportions, along with consistent transport durations, indicates that the program successfully maintained efficiency under varying clinical loads. CABG remained the predominant surgical procedure, especially among emergent patients, emphasizing the program’s responsiveness to ischemic heart disease [12, 13]. The relatively short median time to surgery for emergent cases (4 h) reflects a high level of logistical coordination between the hospitals, from transfer initiation to operative readiness, this factor was crucial to consider in the study, as the duration of the operation is a well-known predictor of outcomes [14, 15]. Telemedicine integration represents the next logical evolution for this program. Real-time virtual consultations can facilitate early triage decisions, optimize patient stabilization prior to transfer, and reduce unnecessary referrals, the efficacy of such programs was also demonstrated in Morh et al. study [16]. Standardized digital documentation and dedicated transfer coordination teams may further streamline operations, decrease time to surgery, and improve outcomes.

The findings of this study provide actionable insights into optimizing IHT programs for cardiac surgical patients. The demonstrated ability to transfer emergent patients with a median time-to-surgery of only 4 h confirms that well-structured interfacility logistics can meet the critical time demands of life-threatening cardiac conditions. This supports the broader implementation of time-targeted transfer protocols, particularly for patients at high risk for preoperative shock or myocardial infarction. Furthermore, the observed trend toward increased urgent referrals over time suggests that program transparency and reliability foster greater trust among referring clinicians—a factor that may enhance early identification and mobilization of at-risk patients. The stable proportion of emergent cases, despite rising volumes, highlights the system’s resilience and scalability. These results argue for wider adoption of standardized triage tools and escalation pathways within regional hospital networks. Integration of telemedicine and dedicated transfer coordinators, as proposed, would not only improve clinical decision-making but also reduce time-to-treatment disparities—an especially relevant factor in geographically distributed healthcare systems.

Notably, several measures could further enhance the IHT process:

- Implementing standardized triage criteria across referring centers [17].
- Establishing dedicated IHT coordinators [18].
- Enhancing pre-transfer stabilization protocols [19].
- Incorporating predictive risk stratification tools to assist with surgical prioritization [20].

## Limitations

Despite promising outcomes, this study is limited by its retrospective nature and the lack of a control group. Variability in institutional practices and surgical expertise may also confound results. Prospective studies should evaluate the impact of real-time data exchange, centralized triage, and expanded regional collaborations on long-term surgical outcomes.

## Data Availability

The underlying data supporting the study’s findings will be provided upon reasonable request to the corresponding author.

## References

[1] Emanuelson RD, Brown SJ, Termuhlen PM. Interhospital transfer (IHT) in emergency general surgery patients (EGS): a scoping review. Surg Open Sci. 2022;9:69–79. 35706931 10.1016/j.sopen.2022.05.004 PMC9190042

[2] Mueller SK, et al. Rates, predictors and variability of interhospital transfers: a national evaluation. J Hosp Med. 2017;12(6):435–42. 28574533 10.12788/jhm.2747 PMC11096839

[3] Murshed I, et al. Surgical inter-hospital transfers: life saver or resource drainer? ANZ J Surg. 2022;92(6):1300–1. 35688641 10.1111/ans.17786 PMC9328366

[4] Chan JC, et al. Nobody told me: communication issues affecting Australian cardiothoracic surgery patients. Ann Thorac Surg. 2019;108(6):1801–6. 31254505 10.1016/j.athoracsur.2019.04.116

[5] Badal BD, et al. Predictors of hospital transfer and associated risks of mortality in acute pancreatitis. Pancreatology. 2021;21(1):25–30. 33341342 10.1016/j.pan.2020.12.001

[6] Lawless AM, et al. Time to surgery and transfer-associated mortality for hip fractures in Western Australia. ANZ J Surg. 2020;90(9):1750–3. 32729649 10.1111/ans.16115

[7] Sanaiha Y, et al. Impact of interhospital transfer on clinical outcomes and resource use after cardiac operations: insights from a National cohort. Surgery. 2020;168(5):876–81. 32641276 10.1016/j.surg.2020.05.026

[8] Metkus TS, et al. Presentation and outcomes of patients with preoperative critical illness undergoing cardiac surgery. JACC: Advances. 2023;2(2):100260. 38357248 10.1016/j.jacadv.2023.100260 PMC10865183

[9] Sinha S, et al. Systematic review and meta-analysis of mortality risk prediction models in adult cardiac surgery. Interact Cardiovasc Thorac Surg. 2021;33(5):673–86. 34041539 10.1093/icvts/ivab151 PMC8557799

[10] Nashef SA, et al. EuroSCORE II. Eur J Cardiothorac Surg. 2012;41(4):734–44. discussion 744-5. 22378855 10.1093/ejcts/ezs043

[11] Newton SM, Fralic M. Interhospital transfer center model: components, themes, and design elements. Air Med J. 2015;34(4):207–12. 26206546 10.1016/j.amj.2015.03.008

[12] Beller JP, et al. Impact of transfer status on real-world outcomes in nonelective cardiac surgery. J Thorac Cardiovasc Surg. 2020;159(2):540–50. 30878161 10.1016/j.jtcvs.2018.12.107 PMC6689463

[13] O’Brien SM, et al. The society of thoracic surgeons 2018 adult cardiac surgery risk models: part 2-Statistical methods and results. Ann Thorac Surg. 2018;105(5):1419–28. 29577924 10.1016/j.athoracsur.2018.03.003

[14] Cheng XF, et al. Risk factors for postoperative myocardial injury-related cardiogenic shock in patients undergoing cardiac surgery. J Cardiothorac Surg. 2023;18(1):220. 37415183 10.1186/s13019-023-02312-3 PMC10326937

[15] Seal F, Wang SH, Zheng B. Identifying the effect of the surgical team on cardiac surgery operation time: a retrospective analysis. Perioper Care Oper Room Manag. 2022;26:100226.

[16] Mohr NM, et al. Emergency department telemedicine shortens rural time-to-provider and emergency department transfer times. Telemed e-Health. 2018;24(8):582–93. 10.1089/tmj.2017.0262 29293413

[17] Reichheld A, et al. Defining best practices for interhospital transfers. J Healthc Qual. 2021;43(4):214–24. 33596008 10.1097/JHQ.0000000000000293

[18] Min HS, et al. Operation of National coordinating service for interhospital transfer from emergency departments: experience and implications from Korea. BMC Emerg Med. 2023;23(1):15. 36765283 10.1186/s12873-023-00782-1 PMC9913013

[19] Herrigel DJ, et al. Interhospital transfer handoff practices among US tertiary care centers: a descriptive survey. J Hosp Med. 2016;11(6):413–7. 27042950 10.1002/jhm.2577 PMC5739590

[20] Lee L, et al. Risk stratification in providing inter-facility transport: experience from a specialized transport team. World J Emerg Med. 2010;1(1):49–52. PMC4129761 25214941

